# Neither slim nor fat: estimating the mass of the dodo (*Raphus cucullatus*, Aves, Columbiformes) based on the largest sample of dodo bones to date

**DOI:** 10.7717/peerj.4110

**Published:** 2017-12-05

**Authors:** Anneke H. van Heteren, Roland C.H. van Dierendonk, Maria A.N.E. van Egmond, Sjang L. ten Hagen, Jippe Kreuning

**Affiliations:** 1 Sektion Mammalogie, Zoologische Staatssammlung München (Staatliche Naturkundliche Sammlungen Bayerns), Munich, Germany; 2 Instituut voor Interdisciplinaire Studies, Universiteit van Amsterdam, Amsterdam, The Netherlands

**Keywords:** Dodo, Mass estimate, Regression method, Femur, Circumference, Log detransformation bias

## Abstract

The dodo (*Raphus cucullatus*) might be the most enigmatic bird of all times. It is, therefore, highly remarkable that no consensus has yet been reached on its body mass; previous scientific estimates of its mass vary by more than 100%. Until now, the vast amount of bones stored at the Natural History Museum in Mauritius has not yet been studied morphometrically nor in relation to body mass. Here, a new estimate of the dodo’s mass is presented based on the largest sample of dodo femora ever measured (*n* = 174). In order to do this, we have used the regression method and chosen our variables based on biological, mathematical and physical arguments. The results indicate that the mean mass of the dodo was circa 12 kg, which is approximately five times as heavy as the largest living Columbidae (pigeons and doves), the clade to which the dodo belongs.

## Introduction

The dodo (*Raphus cucullatus*) was an enigmatic endemic flightless pigeon from the island of Mauritius and its closest extant relative is the Nicobar Ground Pigeon, *Caloenas nicobarica* (Shapiro et al., 2002). The dodo went extinct on Mauritius toward the end of the 17th century as a consequence of human colonization of the island (Hume, Martill & Dewdney, 2004; Cheke, 2006). It had only been discovered a mere 150 years before its extinction and most knowledge about this bird derives from what was written during this period. A number of researchers have estimated its body mass over the years and estimates of its mass vary between 9.5 and 21.2 kg (Table 1). With the higher estimates more than doubling the lower, this range is extraordinarily large. Furthermore, contemporary paintings also depict interpretations of the dodo that vary from meager to fat (Oudemans, 1917), indicating confusion on the topic ever since the discovery of the species. Over time, various explanations have been offered for these discrepancies. Seasonal fluctuations have been postulated (Oudemans, 1917), as well as obesity in captive birds (Kitchener, 1993). Recently, molting has also been proposed as a possible explanation for varying appearances of dodos in art (Angst et al., 2017). Alternatively, however, depictions, particularly by seamen, may also have been less than accurate (Hume, 2006).

**Table 1:** Schematic overview of previous studies.

Study	Birds (number of species)	Elements	Measurement	Dodo mass (*n*)	Remarks
Campbell & Marcus (1992)	All birds (387)	F	Circumference	13.2–16.4 (3)	
Livezey (1993)	Columbidae (187)	F	Length	Male: 10.6 (7)	“Flighted” model
				Female: 8.6 (8)	
				Male: 15.9 (7)	50% addition to correct for flightlessness *or* fat condition
				Female: 12.9 (8)	
				Male: 21.2 (7)	100% addition to correct for flightlessness *and* fat condition
				Female: 17.2 (8)	
Kitchener (1993)	Columbidae (32)	F, TT, TMT	Length, diameter	10.6–17.5 (29 F, 32 TT, 26 TMT)	
Angst, Buffetaut & Abourachid (2011a)	All birds (323)	F, TT, TMT	Length	10.5 (25 F, 27 TT, 30 TMT)	Based on the regression of Zeffer, Johansson & Marmbebro (2003)
Louchart & Mourer-Chauviré (2011)	All birds (387)	F	Circumference	11.7–15.4 (3)	Based on the regressions of Campbell & Marcus (1992)
	Heavy terrestrial birds	F	Circumference	9.5–12.3 (3)	

**Notes:**

Schematic overview of previous studies aiming to estimate the weight of the dodo using the regression method. F, femur; TT, tibiotarsus; TMT, tarsometatarsus; *n*, number of dodo bones used in the study.

In recent years, scientists have developed a variety of methods in order to determine the mass of extinct animals. The non-uniform rational B-splines model and the convex hull approach (Brassey et al., 2016) are excellent examples of modeling techniques. The regression method used in this study is fundamentally different, however. It does not reconstruct the body mass of a single individual based on the shape of the whole skeleton, but it calculates the mass of multiple individuals, when available, based on a mathematical comparison of skeletal measurements with reference taxa. The regression method used in this study is based on the allometric relationship between body mass and bone dimensions in the form of

$$y = c\;{x^b}$$

in which *y* represents body mass, *c* a constant, *x* a bone dimension and *b* the allometric exponent (Damuth & MacFadden, 1990; Garcia & da Silva, 2006). Taking the logarithm of the values, in order to get a linear regression, and solving for *y*, the bird’s body mass, yields:

$$y = {10^{c\; + \;b\;{\rm{log}}\left( x \right)}}$$

The accuracy of such regressions is dependent on three main considerations (Reynolds, 2002): the reference taxa, the bone element and the bone dimension (e.g., length or circumference). Previous attempts to estimate the mass of the dodo using the regression method were based on a variety of assumptions, which perhaps explains the relatively large spread in results (Table 1). In order to make the best choices with regards to these considerations, not only biological, but also mathematical and physical arguments need to be taken into account (Reynolds, 2002). Indeed, it may be argued that the regression method can only lead to a reliable estimate of the mass of the dodo when an interdisciplinary approach is followed.

Estimates of the dodo’s body mass based on regression equations, including means and ranges, have been made five previous times (Table 1). The present study builds on the approaches of these researchers and aims to adopt the best practices from each of them and to apply them to a large number of dodo bones to arrive at, arguably, the most accurate estimate of the dodo’s mean body mass to date. To build the regression models, three main aspects should be taken into consideration, namely which measurement to take on which skeletal element and which species to use in the reference dataset.

The first consideration deals with which of the skeletal elements should be used for the regression analysis. In theory, a multivariate regression based on multiple skeletal elements is likely to have improved predictive power (Serrano, Palmqvist & Sanz, 2015), but the present sample of dodo bones consists primarily of unassociated lower limb bones, which precludes this possibility. Angst, Buffetaut & Abourachid (2011a) used the average of three leg bones (femur, tibiotarsus or tarsometarsus) to determine the mass of the dodo, but these bones were also not associated.

Others have argued that the femur is more representative of bird mass than the other leg bones (Campbell & Marcus, 1992; Livezey, 1993; Louchart et al., 2005; Louchart & Mourer-Chauviré, 2011). In birds, the main function of the femur is to support mass, whereas the other leg bones have additional functions and show morphological variation related to foraging and locomotory behavior (Campbell & Marcus, 1992; Louchart & Mourer-Chauviré, 2011). As an additional physical argument, it should be noted that the femur is positioned relatively horizontally, whereas the tibiotarsus and the tarsometatarsus are positioned more vertically. The horizontal position of the femur increases the moment arm of the point of gravity of the bird’s femur, which is why it is generally assumed that, of all three leg bones, the femur correlates best with body mass (Campbell & Marcus, 1992; Livezey, 1993; Louchart et al., 2005; Louchart & Mourer-Chauviré, 2011). Two recent studies involving many different skeletal measurements also empirically found the femur to be the most accurate predictor of bird body mass (Field et al., 2013; Serrano, Palmqvist & Sanz, 2015).

The second consideration concerns the bone dimensions that should be measured. Both length (Kitchener, 1993; Livezey, 1993; Angst, Buffetaut & Abourachid, 2011a) and some measure of thickness (e.g., circumference, width or breadth) (Campbell & Marcus, 1992; Louchart & Mourer-Chauviré, 2011) have been used by previous authors. Angst, Buffetaut & Abourachid (2011a) used length, which enabled them to use the regression equations of Zeffer, Johansson & Marmbebro (2003). In a response, Louchart & Mourer-Chauviré (2011) pointed out that circumference is a better correlate with mass than length, which they inferred from a higher coefficient of determination (*R*^2^). Campbell & Marcus (1992) preferred circumference over length, arguing that the smallest circumference of the bone would be best correlated to mass, because here the bone is weakest, assuming that there are no differences in bone density along the dodo’s femur.

Femur length depends a lot on other biological factors besides body mass, such as feeding behavior and habitat (Louchart & Mourer-Chauviré, 2011). Two recent studies on regression methods, using extant flying bird datasets for body mass estimations of fossil flying birds, elaborate on the choice of bone and bone dimension. Field et al. (2013) calculated the percent prediction error (PPE), the scaled difference between predicted and observed body mass, for a number of skeletal measurements. The available dodo bones include only leg bones and, therefore, only those are relevant here. Of the leg elements, the circumference of the femur represented the strongest correlation with body mass (*R*^2^ = 0.95) and showed the smallest prediction error (Mean PPE = 32.79). Figure 3 in Field et al. (2013) also clearly shows that, of the leg bone measurements, femur circumference performs better than femoral diameter, femoral length, or measurements on the tarsometatarsus or tibiotarsus. Serrano, Palmqvist & Sanz (2015) found that the mediolateral breadth of the femur correlates strongest with body mass (*R*^2^ = 0.95272), but they did not include circumference, which would be expected to have correlated even better (Field et al., 2013). Since bird bones are essentially hollow tubes (Biewener, 1982), it may be argued that cortical thickness also has an appreciable effect on the strength of the bone (Habib & Ruff, 2008; Habib, 2010) and consequently might be related to body mass. Cortical thickness is, however, only measurable by radiographic investigation or sectioning the bone, which is outside the scope of this study. Femur circumference is, therefore, the most appropriate measurement currently available.

The third consideration involves which taxa are most appropriate to compare the dodo with. Some birds weigh only several grams, while other (extinct) birds have reached body masses of hundreds of kilograms (Marchant & Higgins, 1990; Del Hoyo, Elliot & Sargatal, 1992, 1996; Higgins & Davies, 1996; Del Hoyo, Elliot & Sargatal, 1997). To estimate the mass of the dodo, there are roughly three different approaches for selecting the reference taxa; one could opt to compare the dodo with its closest relatives (i.e., Columbidae), birds with similar life-styles (e.g., other flightless birds) or with as many birds as possible, the latter increasing statistical accuracy, but forgoing information on phylogeny and life-style.

Livezey (1993) and Kitchener (1993) calculated dodo masses using regression analyses that were only based on Columbidae, being the closest living relatives of the dodo. Livezey (1993) thought this would underestimate the mass of the dodo and corrected for this by adding 50%. Angst, Buffetaut & Abourachid (2011a) used the regression analyses of Zeffer, Johansson & Marmbebro (2003), which included hundreds of bird species, to maximize statistical accuracy. Campbell & Marcus (1992) created several regression equations with functional subsets of bird species, including heavy-bodied terrestrial birds, but chose to use the regression analysis including all birds to estimate the mass of the dodo. Louchart & Mourer-Chauviré (2011), however, did choose to use the heavy-bodied terrestrial birds regression equation of Campbell & Marcus (1992), reasoning that these animals both have a similar build to the dodo and include its closest living relatives.

To estimate the body mass of extinct species, it is imperative that a reasonable analog for the fossil animals is chosen, which is based on affinity, whether phylogenetic, functional or a combination (Reynolds, 2002). As the dodo is much larger than the largest living columbid (*Goura victoria*, 2.5 kg), basing the regression analysis on the dodo’s relatives only would inevitably mean having to extrapolate to find the mass of the dodo. From a mathematical point of view, it is clear that this would be less accurate than interpolation, since both the confidence (CI) and the prediction (PI) intervals become wider as the target value of the independent variable moves away from the mean and a mathematical model is never absolutely correct (Wonnacott & Wonnacott, 1990).

The dodo is a large flightless bird. Therefore, comparison with other heavy terrestrial birds would be ideal. Until now, no dataset is available that includes skeletal measurements and bird masses of large flightless birds, such as the larger ratite species. Given the dodo’s ancestry and locomotory habits, however, a dataset consisting of heavy-bodied terrestrial birds as defined by Campbell & Marcus (1992) would be a reasonable alternative. This dataset would, nonetheless, require extrapolation to estimate the body mass of the dodo. And, Campbell & Marcus (1992) only provide the resultant equations, but not the raw data, making it difficult to reuse their results in new analyses.

Alternatively, it might be an option to select a similar group of heavy-bodied terrestrial birds from the large dataset of Field et al. (2013). In the Field et al. (2013) dataset, only five of the families belonging to the heavy-bodied terrestrial birds group of Campbell & Marcus (1992) are available: Columbidae, Phasianidae, Cracidae, Tetraonidae and Numididae. The families used by Field et al. (2013) are primarily geared toward flighted birds (Table 2), whereas Campbell & Marcus (1992) also used Tinamidae, Apterygidae and Turnicidae, which are either flightless or avoid flight. The latter are omitted in the Field et al. (2013) dataset. As the paper of Field et al. (2013) is especially geared toward modern and fossil flying birds, this should come as no surprise and, in fact, implies that they have very carefully selected their reference taxa to suit their research questions. Taking such a subset from Field et al. (2013) would be inappropriate for the research question at hand here, since the present paper is aimed at a flightless species. We will follow the example of Field et al. (2013), as well as the recommendation of Reynolds (2002), and carefully select our reference taxa to suit our research goal, which is to estimate the average mass of the dodo, and the range of masses as displayed by a natural population of dodos, in order to contribute to solving the mystery of the mass of the dodo.

**Table 2:** Comparison of bird families used in different analyses.

Family	Locomotory habit
Tinamidae	Avoid flight
Apterygidae	Flightless
Anhimidae	Flighted
Cracidae	Flighted
Numinidae	Flighted
Phasianidae	Avoid flight
Tetraonidae	Flighted
Turnicidae	Avoid flight
Pteroclidae	Flighted
Columbidae	Flighted

**Notes:**

Families used by Campbell & Marcus (1992) in their analysis of heavy-bodied terrestrial birds and a categorization of their locomotory habits. Red indicates the families that are not represented in Field et al. (2013).

## Materials

The majority of dodo bones that are preserved worldwide come from Mare aux Songes. This is a shallow swamp in the southeast of Mauritius, which includes a bone bed consisting primarily of tortoise bones (Rijsdijk et al., 2009). Radiocarbon dates indicate that the fossils were deposited in a relatively short time frame, between 4,235 and 4,100 years ago (Rijsdijk et al., 2009, 2011, 2015). Most dodo bones were excavated during the 19th century and are stored in the Natural History Museum of Mauritius in Port Louis and owned by the Mauritius Museums Council, but many can also be found in various museums throughout Europe. The vast amount of bones stored at the Natural History Museum of Mauritius has been studied morphologically (Janoo, 1997), but not yet morphometrically or in relation to body mass.

In total, 174 dodo femora were measured (82 right and 92 left bones), which is currently the largest sample of dodo femora ever measured (Supplemental Information 1). All 174 dodo bones used in the analyses presented here derive from the Mare aux Songes on Mauritius. They are stored at the Natural History Museum of Mauritius (Mauritius Museums Council), OmniCane, Natural History Museum London, Natural History Museum at Tring, Muséum national d’Histoire naturelle, Naturalis Biodiversity Center and the University Museum of Zoology Cambridge. The comparative reference dataset consists of eight flightless ratites, 19 medium-sized primarily ground-dwelling birds (Galloanserae and Tinamiformes, as opposed to the heavy-bodied terrestrial birds of Campbell & Marcus (1992)) and 16 Columbidae. Skeletal measurements were collected at Naturalis Biodiversity Center, in combination with body mass data from the literature (Gibbs, Barnes & Cox, 2001; Marchant & Higgins, 1990; Del Hoyo, Elliot & Sargatal, 1992, 1996, 1997).

## Methods

**Figure 1:**
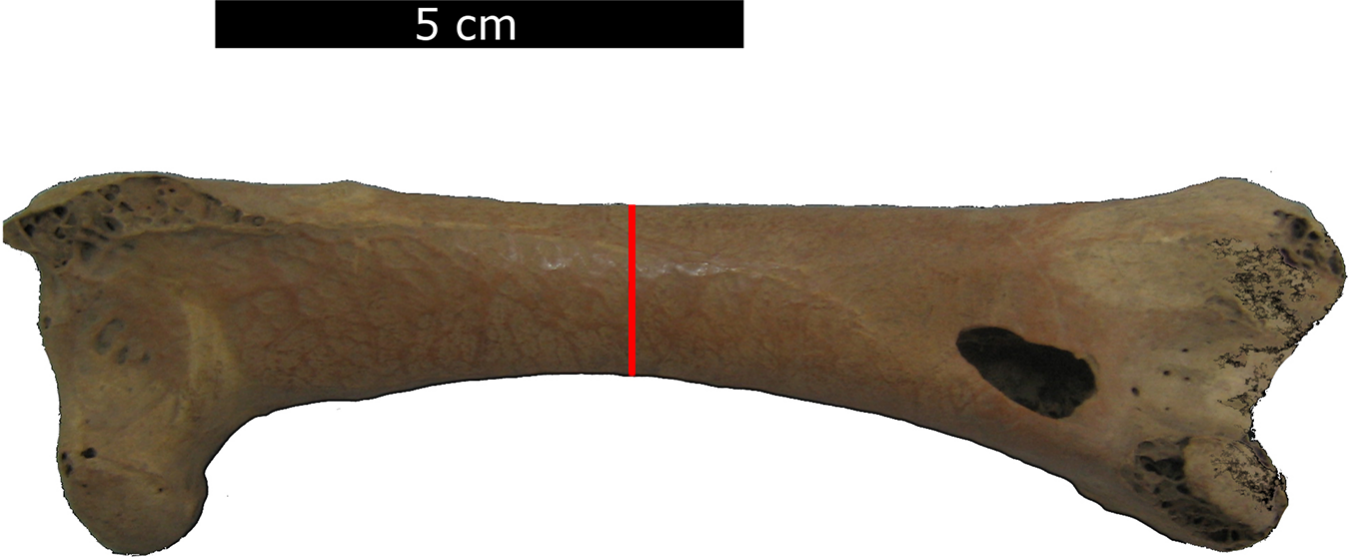
Measurement position. The position of the circumference measurement on the femur as indicated by the red line.

In dodos, the thinnest circumference of the femur coincides with mid-shaft (Fig. 1). The circumference of the femur at mid-shaft of dodos and reference taxa was measured with a tape measure to an accuracy of 1 mm (Supplemental Information 1 for dodos and 2 for reference taxa).

The left and right bones were tested for normality and homogeneity of variance, before performing an independent samples *t*-test for differences between left and right bones. The coefficient of variation (CV) was calculated following Halgrímsson & Maiorana (2000):

$${\rm{CV}} = 100s/\bar X$$

in which *s* is the sample standard deviation, and $\bar {X}$ is the sample mean.

Femora of Columbidae (closest living relatives), Galloanserae and Tinamiformes (medium-sized ground-dwelling birds) and Ratites (flightless birds) were measured (Supplemental Information 2). The regressions were performed on various combinations of these groups to determine what influence the choice of reference taxa has on the resultant regression equations.

The present reference dataset is not very large, so comparisons with existing datasets and regression equations are warranted. Campbell & Marcus (1992) provide the following regression equation:

$${\rm{BM}} = {10^{c\; + \;b\;{\rm{log}}\left( {{\rm{FC}}} \right)}}$$

in which BM = body mass, FC = femur circumference. Taking into account considerations of evolutionary relationship and life-style, the regression of Campbell & Marcus (1992) based on heavy-bodied terrestrial birds, including Columbiformes and Galliformes (cf. Louchart & Mourer-Chauviré, 2011), would be an appropriate equation to estimate the mean body mass of the dodo. In that case the following parameters would apply: *c* = 0.110 and *b* = 2.268^1^1Additional information regarding Campbell & Marcus’ (1992) regression using heavy-bodied terrestrial birds (HB): *N* species = 39, *N* specimen = 82, *R*^2^ = 0.969, 95% CI of the slope (rma): [2.131, 2.413]. The authors do not provide an *F* value or the 95% CI for the *Y*-intercept.. For the regression equation using all birds, the following parameters apply: *c* = −0.118 and *b* = 2.463^2^2Additional information regarding Campbell & Marcus’ (1992) regression using all birds (AL): *N* species = 387, *N* specimen = 795, *R*^2^ = 0.961, 95% CI of the slope (rma): [2.415, 2.512]. The authors do not provide an *F* value or the 95% CI for the *Y*-intercept.. The reference dataset of Field et al. (2013) might also provide an alternative. Field et al. (2013) found the following relationship between femur circumference (FC) and body mass (BM) with *R*^2^ = 0.95 and mean PPE = 32.79:

$${\rm{ln}}\left( {{\rm{BM}}} \right) = 2.40\left( {{\rm{ln\;FC}}} \right)$$

When performing regressions on log transformed data, a bias occurs when detransforming the data to their original scale (Smith, 1993). Serrano, Palmqvist & Sanz (2015), for example, have corrected for this bias by applying the ratio estimator by Snowdon (1991). Clifford et al. (2013) review nine correction factors that one might use to correct for this bias. They recommend using a method by Shen & Zhu (2008), which is designed to minimize mean squared error, when predicting the biomass of new trees. Birds, however, are not trees and, whereas data in forestry appears to often be normally distributed (Clifford et al., 2013), this is certainly not the case in birds, where there are many smaller species and only a few larger ones. Therefore, all nine correction factors were tested on the complete reference dataset provided by Field et al. (2013), as well as the data presented here. The mean PPE was used as a measure of how well each correction factor performed.

Both Campbell & Marcus (1992) and Field et al. (2013) provide regression equations for femur circumference. Campbell & Marcus (1992), however, do not provide their raw data, thereby making it impossible to calculate a log detransformation bias correction factor. Nevertheless, the analyses were performed using both the equations provided by Campbell & Marcus (1992) and the dataset of Field et al. (2013), so that the outcomes could be compared with the regression equations based on the present dataset. Using the function *predict* (Chambers & Hastie, 1992), PIs were calculated in the R stats package and plotted in Microsoft Excel.

## Results

There is no significant difference between the circumference of the left and right femora (*t*(172) = −1.586, *p* = 0.115). Therefore, in order to increase sample size and statistical power, the entire dataset was analyzed together.

Then a correction for the log detransformation bias was chosen empirically. Although all the mean PPEs are fairly similar per dataset (Table 3), the results show that the ratio estimator (Ratio) by Snowdon (1991) is generally the worst performing ratio estimator in three out of five tested datasets. In the cases of the subset of Field et al. (2013) and the data presented here without Columbidae (i.e., medium-sized ground-dwelling and flightless birds), it performed even worse than not performing a correction at all. For the dataset consisting of ratites only, the ratio estimator was, however, the best performing correction. The smearing estimate (Smear) by Duan (1983) performs best in four out of five datasets. Since there is no correction that consistently performs best, all correction factors were tested and the best performing one chosen before any mass estimates of the dodo were made.

**Table 3:** Comparison of various log detransformation bias correction factors.

Correction factor	Field et al. (2013) all birds	Field et al. (2013) subset	Present data full dataset	Present data heavy and flightless	Present data flightless
No correction factor	32.78883	21.05430	23.31936	18.25612	12.48232
REML	31.89988	21.04287	23.08221	18.15626	12.27288
ML	31.90159	21.05430	23.0887	18.15897	12.29152
Finney’s	31.90025	21.04537	23.08363	18.15685	12.27639
Ratio	32.15498	22.15854	22.92239	18.87176	11.32922
UMVU	Inf^1^	21.20675	23.21062	18.21409	12.40849
EV	31.90125	21.06107	23.09212	18.16617	12.31257
MM	31.90565	21.09565	23.11133	18.17999	12.3658
MB	31.90124	21.06022	23.09162	18.16552	12.30875
Smear	31.12007	20.75643	22.93455	18.15139	12.12568

**Notes:**

The mean percent prediction error of the naive estimate (no correction factor) and nine different correction factors using all birds and a subset of the data of Field et al. (2013), as well as various combinations of the data presented herein. Abbreviations for the correction factors follow Clifford et al. (2013). Worst and best performing correction factors are indicated in red and green respectively.

1 The calculation of this correction factor returns “Inf” (=infinite),when the sample size exceeds a certain threshold, using the formula for the UMVU correction provided by Clifford et al. (2013), which is based on the hyperg_0F1 function in the gsl package in R.

**Figure 2:**
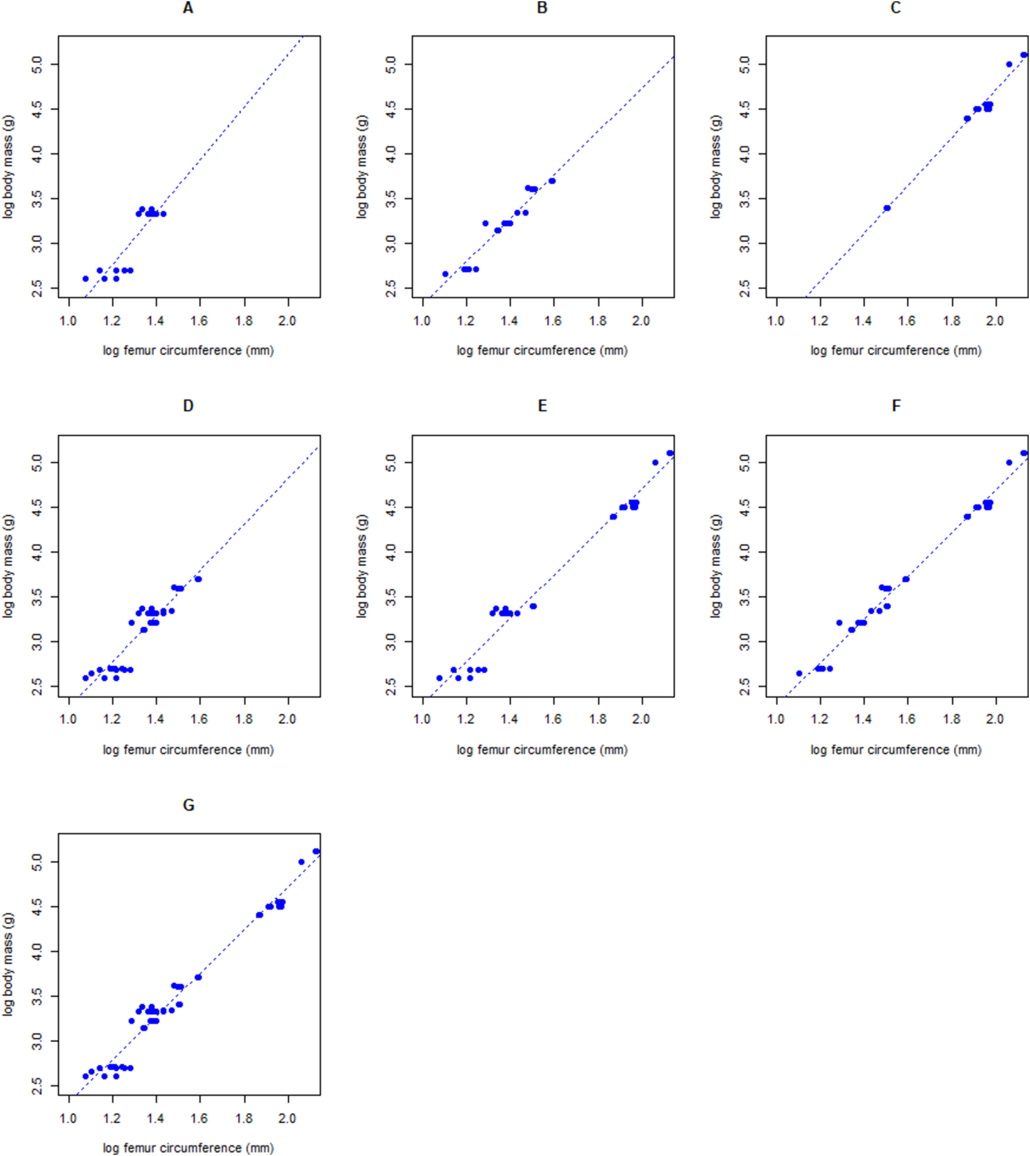
Regression results. Plots with linear regression lines for log femur circumference (mm) on the horizontal axis against log body mass (g) on the vertical axis for various subsets of birds; (A) Columbidae (close relatives of the dodo), (B) Galloanserae and Tinamiformes (medium-sized ground-dwelling), (C) Ratites (flightless), (D) Columbidae and medium-sized ground-dwelling birds, (E) Columbidae and flightless birds, (F) medium-sized ground-dwelling and flightless birds, (G) All groups combined.

Regressions of log body mass onto log femur circumference were performed for each combination of the three bird groups (Fig. 2). The results of the regressions are very similar to each other with similar intercepts and slopes, as well as relatively high *R*^2^’s (Table 4). In fact, the *R*^2^ of the combined regression (G in Table 4) is only 0.011 lower than the highest *R*^2^ displayed by the flightless ratites (C in Table 4) or the ratites in combination with medium-sized ground-dwelling birds (F in Table 4). The PPE is lowest in flightless Ratites alone. The *R*^2^ is the highest in Ratites only and the combined group of Ratites with medium-sized ground-dwelling birds. Based on these results, dodo body masses were calculated based Ratites only, and Ratites and medium-sized ground-dwelling birds, as well as the full dataset presented here, the dataset of Field et al. (2013) and the regressions of Campbell & Marcus (1992).

**Table 4:** Linear regression results.

	Categories included	*N*	Slope	Intercept	*R*^2^	PPE naive	PPE corr.
A	Columbidae (closest relatives)	16	2.9462	−0.7754	0.796	30.1	28.3
B	Galloanserae and Tinamiformes (heavy-bodied)	19	2.4414	−0.1412	0.933	18.1	18.1
C*	Ratites (flightless)	18	2.67942	−0.63748	0.985	12.5	11.3
D	Columbidae, Galloanserae and Tinamiformes	35	2.5660	−0.2978	0.871	25.2	24.5
E*	Columbidae, Ratites	34	2.4241	−0.1313	0.974	26.5	25.7
F	Galloanserae and Tinamiformes, Ratites	37	2.42748	−0.14354	0.985	18.3	18.2
G	Columbidae, Galloanserae and Tinamiformes, Ratites	53	2.41731	−0.11616	0.974	23.3	22.9

**Notes:**

Results of the linear regression lines for log femur circumference (mm) against log body mass (g) for various combinations of bird groups. Columbidae are the closest living relatives, Galloanserae and Tinamiformes are medium-sized ground-dwelling birds and Ratites are flightless. The letters correspond to those in Fig. 2. Percent prediction error (PPE) for the naive estimate are given in addition to the coefficient of variation (*R*^2^). Categories with an * were corrected using the ratio estimator, all other categories using the smearing coefficient.

The various dodo mean body mass estimates are summarized in Table 5, based on the individual mass estimates provided in Supplemental Information 1. Using all birds of the reference dataset presented here and applying the smearing estimate, we find a mean body mass of 14.1 kg for the dodo with a 95% CI between 13.8 and 14.4 kg. When the regression on heavy-bodied semi-terrestrial and flightless birds is used in combination with the smearing estimate, the mean body mass of the dodo is calculated to be slightly lower at 13.6 kg with a 95% CI between 13.3 and 13.8 kg. When the regression based on only flightless birds is used in combination with the ratio estimator, the mean mass of the dodo is again estimated to be slightly lower at 12.4 kg with a CI between 12.1 and 12.6 kg and a range of 7.7–18.2 kg.

**Table 5:** Dodo body mass estimates.

Present data all birds	Naive estimate	Smearing estimate	Field et al. (2013) all birds	Naive estimate	Smearing estimate
Minimum	8.8	9.2	Minimum	9.5	10.3
Maximum	19.2	19.9	Maximum	20.5	22.1
1. Quartile	12.3	12.8	1. Quartile	13.2	14.3
3. Quartile	14.6	15.1	3. Quartile	15.6	16.9
Mean	13.5	**14.1**	Mean	14.5	**15.7**
Median	13.4	14.0	Median	14.4	15.5
LCL mean	13.3	13.8	LCL mean	14.2	15.4
UCL mean	13.8	14.3	UCL mean	14.8	16.0
Variance	3.1	3.4	Variance	3.5	4.1
CV	13.1	13.1	CV	12.9	12.9

Present data heavy-bodied and ratites	Naive estimate	Smearing estimate	Campbell & Marcus (1992)	All birds Naive estimate	
Minimum	8.6	8.8	Minimum	10.5	
Maximum	18.8	19.2	Maximum	23.1	
1. Quartile	12.1	12.4	1. Quartile	14.7	
3. Quartile	14.3	14.6	3. Quartile	17.5	
Mean	13.2	**13.6**	Mean	**16.2**	
Median	13.1	13.5	Median	16.1	
LCL mean	13.0	13.3	LCL mean	15.9	
UCL mean	13.5	13.8	UCL mean	16.5	
Variance	3.0	3.1	Variance	4.6	
CV	13.1	13.1	CV	13.3	

Present data only flightless	Naive estimate	Ratio estimate	Campbell & Marcus (1992)	Subset Naive estimate	
Minimum	7.4	7.7	Minimum	8.4	
Maximum	17.3	18.2	Maximum	17.2	
1. Quartile	10.6	11.1	1. Quartile	11.4	
3. Quartile	12.8	13.4	3. Quartile	13.4	
Mean	11.8	**12.4**	Mean	**12.5**	
Median	11.7	12.3	Median	12.4	
LCL mean	11.5	12.1	LCL mean	12.2	
UCL mean	12.0	12.6	UCL mean	12.7	
Variance	2.9	3.2	Variance	2.3	
CV	15.5	14.4	CV	12.2	

**Notes:**

Statistics summaries for dodo body mass estimates calculated using three regressions on the data presented here (left), and three on the literature (right) (Campbell & Marcus, 1992; Field et al., 2013). The important means are indicated in bold. The naive estimates are also given to allow for a direct comparison with the regressions of Campbell & Marcus (1992), for which only the naive estimates are available.

Using the complete reference dataset of Field et al. (2013) and the smearing estimate, the mean dodo mass is determined to be 15.7 kg with a 95% CI of between 15.4 and 16.0 kg and a range of 10.3–22.1 kg. Using the regression equations of Campbell & Marcus (1992), only the naive estimates can be calculated, since the original data are not available. The regression equation for all birds, by Campbell & Marcus (1992) which was also used by Louchart & Mourer-Chauviré (2011), provides a mean body mass for the dodo of 16.2 kg with a CI between 15.9 and 16.5 kg. The naive estimate, based on the heavy-bodied terrestrial bird equation of Campbell & Marcus (1992), puts the mean dodo mass at 12.5 kg with a 95% CI of the mean between 12.2 and 12.7 kg and a range of 8.4–17.9 kg.

**Figure 3:**
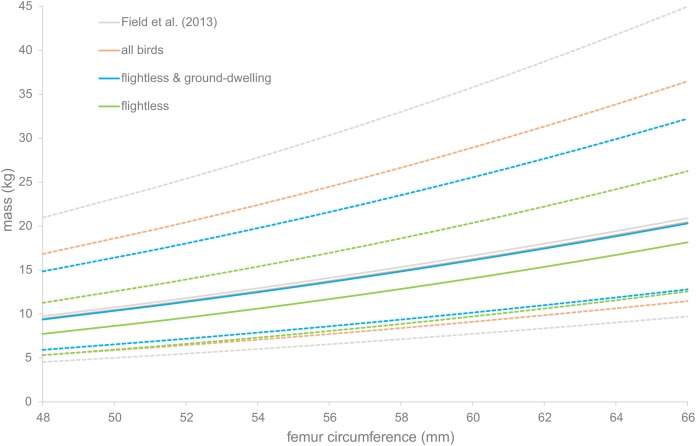
Prediction intervals for dodo mass estimates. Prediction intervals of dodo mass estimates based on different regressions, based on the data of Field et al. (2013) (gray) and the data presented here, including all birds (orange), flightless and medium-sized ground-dwelling birds (blue) and flightless birds (green). Solid lines indicate the estimated body masses and dotted lines the prediction intervals.

The PIs were calculated for four of the regression equations. They are displayed in Fig. 3. For all equations, the PI becomes larger as the bones become larger. The regression based on flightless birds only gives a PI of between 7 and 32 kg for the median specimen, whereas the smallest specimen has a PI of only between 5 and 21 kg.

The CVs were calculated for all regressions except those of Campbell & Marcus (1992). For the reference dataset presented here, including and excluding Columbidae, the CV is 13.1 (Table 5). When only the flightless birds are included in the dataset, the CV is 14.4. For the dataset of Field et al. (2013), it is 12.9. And for the regressions by Campbell & Marcus (1992), for the subset and the full dataset, the CVs are 12.2 and 13.3 respectively.

## Discussion

This paper set out to estimate the mean mass of the dodo, as well as the range of body masses displayed by a natural population. This has been attempted before (Campbell & Marcus, 1992; Kitchener, 1993; Livezey, 1993; Louchart et al., 2005; Angst, Buffetaut & Abourachid, 2011a; Louchart & Mourer-Chauviré, 2011), but with smaller sample sizes and varying results. Additionally, the importance of correcting for the log detransformation bias had not yet been recognized at those times. Here, the body mass of the dodo is estimated based on the largest sample of dodo bones to date, while correcting for the log detransformation bias.

There are several possible formulae that allow for a log detransformation bias correction (Clifford et al., 2013). We have tested nine of those on the reference data presented here and the dataset of Field et al. (2013). The ratio estimator performs worst in three out of five cases (Table 3), but interestingly best for the dataset consisting of Ratites only. It seems, therefore, that the homogeneity of the data and/or the sample size may influence which correction factor performs best. In homogeneous and/or small datasets, the ratio estimator might be the best tool to use. In larger datasets that consist of many different taxa, the smearing estimate appears to be the method of choice.

There are several regression equations from the literature that could be used to estimate the mass of the dodo (Campbell & Marcus, 1992; Field et al., 2013; Serrano, Palmqvist & Sanz, 2015). Instinctively, one might want to use a regression analysis that includes both the closest living relatives (Columbidae) and large terrestrial birds (Ratites), since only then both biological and mathematical constraints are taken into account. Field et al. (2013), however, determined that there is relatively little phylogenetic dependence in their dataset and Serrano, Palmqvist & Sanz (2015) also argue that phylogeny does not play an essential role in the scaling patterns of major limb bones. This points to a primary functional signal in femur dimensions, obviating the need to include Columbidae in the analyses.

Based on the data presented here, the conclusion was reached that the choice of taxa has a large effect on the results (Table 4). Consequently, choosing the appropriate taxa for the question at hand is very important, possibly more important than maximizing the sample size. In the data presented here, we clearly see that the estimated body mass of the dodo increases as more flighted birds are added to the reference dataset (Table 4). The calculations based on Field et al. (2013), the regression presented here with all birds included and the regression of Campbell & Marcus (1992) on all birds are all approximately one-fifth heavier than those based on the Campbell & Marcus (1992) subset with heavy-bodied terrestrial birds and the regression presented here based on flightless birds only. Our calculations based on flightless together with medium-sized ground-dwelling birds provided intermediate values. This is likely due to the fact that the reference dataset used by Field et al. (2013) and those for the other equations using all birds are primarily based on flying birds. In flying birds, the legs are only used to support weight part-time and it is conceivable that the legs are comparatively thin to contribute to a relatively light build suitable for flying. In flightless birds, there are no, or very little, constraints on total body weight and the legs are continually weight-bearing. As such it is conceivable that the legs of flightless birds are relatively more robust, which has also been shown in the Galapagos cormorant (*Phalacrocorax harrisi*) by Habib & Ruff (2008). Additionally, the regression equation based on the full dataset of Field et al. (2013) results in the largest PI, possibly because the birds in the full dataset represent a multitude of modes of life. As such, the dodo mass estimate based on flightless birds is probably the most accurate. This is corroborated by the fact that this regression has the lowest PPE and the highest *R*^2^.

The estimated mean masses of the dodo are well within the range found by previous studies (Table 1). The most reliable estimates, however, are calculated using the regression on flightless birds in combination with the ratio estimator, which is supported by a smaller PPE, a higher *R*^2^ and a narrower 95% PI. Using this equation, the highest mass estimate of 21 kg for males by Livezey (1993) is not attained by any of the dodos in the large sample presented here and his estimate for females is at the higher end. It appears that his 100% mass addition to correct for flightlessness and a fat condition resulted in overestimates. Without this correction his mean body mass estimates would have been 8.6 and 10.8 kg, which would have been comparable to the results of Angst, Buffetaut & Abourachid (2011a).

The estimate of Angst, Buffetaut & Abourachid (2011a) seems to be very low, which is possibly due to the fact that they used length rather than circumference, but might also be caused by including skeletal elements that are less strongly correlated to body mass than the femur (Louchart & Mourer-Chauviré, 2011). Louchart & Mourer-Chauviré (2011) corrected the estimate of Angst, Buffetaut & Abourachid (2011a) by using the same regression equation of Campbell & Marcus (1992) as used herein. Both ranges provided by them are just below the mean that we calculated using the same equations, which is most likely due to the small sample size of dodo bones (*n* = 3) in their analyses. The highest estimate of their largest individual, using the equation based on the subset of Campbell & Marcus (1992) is very close to our estimated mean. The estimate by Campbell & Marcus (1992) of 13.2–16.4 kg, on the other hand, seems rather high compared to our estimate based on flightless birds. This is probably caused by the fact that they used their regression equation based on all birds; whereas using heavy-bodied terrestrial birds would have been more appropriate for functional reasons (i.e., predominant terrestrial behavior), and would have resulted in a much lower estimate (Table 5). In addition, their sample size also only consisted of three femora. Nevertheless, their range does just overlap with the mean calculated herein using the same regression equation.

The estimates presented here (i.e., a range of 7.7–18.2 kg and a mean of 12.4 kg) are most similar to those of Kitchener (1993), who based his regression analysis on Columbidae only and used a reasonably large sample (*n* = 87) to come to his conclusion, but did not correct for the log detransformation bias.

There is an interesting difference between the estimated body masses presented here and those previously calculated, even when using the same equations. Although the results tend to agree between studies with large sample sizes, studies with smaller sample sizes tend to have much lower or much higher estimates. As there is an inverse square root relationship between the CI of the mean and sample size, the differences between the results presented here and previous estimates emphasize the need for large sample sizes when making generalizing statements about entire species. In fact, one might argue that a large sample size is even more important than the precise composition of the reference dataset or correcting for log detransformation bias.

Angst, Buffetaut & Abourachid (2011b) found, unlike Livezey (1993) and Louchart & Mourer-Chauviré (2011), that the dodo displayed a uniformity in size. Herein, it was tested whether the present data conformed more with the conclusions of Livezey (1993) and Louchart & Mourer-Chauviré (2011) or with Angst, Buffetaut & Abourachid (2011b). Based on the calculations of Halgrímsson & Maiorana (2000), the expected CV for a 12.4 kg bird would be approximately 10. The CV of the reconstructed dodo masses is 14.4 based on the flightless reference dataset presented here, but even the lowest estimate based on the subset of Campbell & Marcus (1992) is 12.2. This is 25–50% higher than expected, which is noteworthy, particularly when taking into account that other Columbidae have rather low CVs for their size (Halgrímsson & Maiorana, 2000). As such, the data presented here support the conclusions of Livezey (1993) and Louchart & Mourer-Chauviré (2011) that the size range displayed by a natural population of dodos is rather wide and implies considerable individual variation and/or sexual dimorphism. A large size range is also very common in insular vertebrates (van der Geer et al., 2010). For example, metatarsal length varies by almost a factor 2 in each of two deer species (*Cervus* sp.) from Malta and from the Ryukyu Islands (J. de Vos, 2015, personal communication; de Vos, 2006). It has, however, not yet been document in insular birds. Both in insular mammals and in the dodo, a lack of mammalian predators may have resulted in relaxed selection pressure on body size. Future research might be able to elucidate whether this pattern is as common in insular birds as in insular mammals.

## Conclusion

There has been a vibrant discussion in the literature on the body mass of the dodo (Campbell & Marcus, 1992; Kitchener, 1993; Livezey, 1993; Louchart et al., 2005; Angst, Buffetaut & Abourachid, 2011a; Louchart & Mourer-Chauviré, 2011). Using the largest sample of dodo femora measured to date, a body mass range between 8 and 18 kg with a mean of 12 kg was estimated herein. This is approximately five times larger than the largest extant columbid (*G. victoria*, 2.5 kg) and similar to a previous estimate by Kitchener (1993). It was also found that the dodo displays increased size variability compared to other columbids, which is a rather common feature in insular mammals, but had not yet been reported for insular birds.

## Supplemental Information

10.7717/peerj.4110/supp-1 Supplemental Information 1 Supplementary Information 1: Raw length and circumference measurements on 174 dodo femora. Raw circumference measurements on 174 dodo femora, as well as mass estimates based on various regressions. Details to abbreviations can be found in the “legend” tab.

10.7717/peerj.4110/supp-2 Supplemental Information 2 Supplementary Information 2: Raw data for the modern birds. Raw circumference measurements on the modern birds. Details on abbreviations can be found in the “legend” tab.

## Additional Information and Declarations

### Data Availability

The following information was supplied regarding data availability: The raw measurements are provided in the Supplemental Information Files.
