# Correction: Neither slim nor fat: estimating the mass of the dodo (*Raphus cucullatus*, Aves, Columbiformes) based on the largest sample of dodo bones to date

**DOI:** 10.7717/peerj.4110/correction-1

**Published:** 2017-12-20

**Authors:** Anneke H. van Heteren, Roland C.H. van Dierendonck, Maria A.N.E. van Egmond, Sjang L. ten Hagen, Jippe Kreuning

**Affiliations:** 1 Sektion Mammalogie, Zoologische Staatssammlung München (Staatliche Naturkundliche Sammlungen Bayerns), Munich, Germany; 2 Instituut voor Interdisciplinaire Studies, Universiteit van Amsterdam, Amsterdam, The Netherlands

Corrigendum: 

After publication the authors noticed that one co-author’s name was listed incorrectly. The author list and author contributions have been amended so that Roland C.H. van Dierendonk is now listed as Roland C.H. van Dierendonck.

